# Development and validation of a web-based nomogram for acute kidney injury in acute non-variceal upper gastrointestinal bleeding patients

**DOI:** 10.3389/fmed.2024.1474311

**Published:** 2024-10-03

**Authors:** Chaolian Wei, Honghua Cao, Lina Huang, Lu-Huai Feng

**Affiliations:** ^1^Department of Gastric and Abdominal Tumor Surgery, Guangxi Medical University Cancer Hospital, Nanning, China; ^2^Department of Gastrointestinal Surgery, Guigang People’s Hospital, Guigang, China; ^3^Department of Endocrinology and Metabolism Nephrology, Guangxi Medical University Cancer Hospital, Nanning, China

**Keywords:** acute kidney injury, prediction, nomogram, intensive care unit, upper gastrointestinal bleeding

## Abstract

**Background:**

Acute kidney injury (AKI) is a common and serious complication in patients with acute non-variceal upper gastrointestinal bleeding (NVUGIB). Early prediction and intervention are crucial for improving patient outcomes.

**Methods:**

Data for patients presenting with acute NVUGIB in this retrospective study were sourced from the MIMC-IV database. Patients were randomly allocated into training and validation cohorts for further analysis. Independent predictors for AKI were identified using least absolute shrinkage and selection operator regression and multivariable logistic regression analyses in the training cohort. Based on the logistic regression results, a nomogram was developed to predict early AKI onset in acute NVUGIB patients, and implemented as a web-based calculator for clinical application. The nomogram’s performance was evaluated through discrimination, using the C-index, calibration curves, and decision curve analysis (DCA) to assess its clinical value.

**Results:**

The study involved 1082 acute NVUGIB patients, with 406 developing AKI. A multivariable logistic regression identified five key AKI predictors: CKD, use of human albumin, chronic liver disease, glucose, and blood urea nitrogen. The nomogram was constructed based on independent predictors. The nomogram exhibited robust accuracy, evidenced by a C-index of 0.73 in the training cohort and 0.72 in the validation cohort. Calibration curves demonstrated satisfactory concordance between predicted and observed AKI occurrences. DCA revealed that the nomogram offered considerable clinical benefit within a threshold probability range of 7% to 54%.

**Conclusion:**

Our nomogram is a valuable tool for predicting AKI risk in patients with acute NVUGIB, offering potential for early intervention and improved clinical outcomes.

## Introduction

Acute non-variceal upper gastrointestinal bleeding (NVUGIB) is a common and serious condition frequently observed in intensive care units (ICU), resulting in substantial morbidity and mortality rates. The in-hospital mortality rate following acute NVUGIB typically ranges from 5% to 15%, but may escalate to 35% among elderly patients with acute kidney injury (AKI) (1). Approximately 5% of hospitalized patients suffer an AKI, with an incidence of 30 - 57% in intensive care units (2), with the average pooled mortality rate of 23% but reached 49.4% in those requiring g kidney replacement therapy (3, 4). Existing literature indicates that AKI occurs in 1–11.4% of patients with acute NVUGIB, and those with acute NVUGIB complicated by AKI have longer hospital stays and higher mortality rates (1, 5). Therefore, early identification of high-risk patients is critical for the prevention of AKI, and early diagnosis and treatment can improve the long-term prognosis of patients (6).

The current diagnostic criteria for AKI as outlined by the Kidney Disease Improving Global Outcomes (KDIGO), an increase in serum creatinine or a decline in urine output remains its key diagnostic criteria (7). However, current clinical detection methods, which rely on creatinine levels and urine output, are inadequate for early AKI diagnosis, AKI is rarely diagnosed and mild cases are often missed (8, 9). In recent years, significant progress has been made in the early diagnosis of AKI due to advancements in information technology, nanotechnology, and biomedicine. Although certain studies have proposed alternative biomarkers—such as cystatin C, neutrophil gelatinase-associated lipocalin, kidney injury molecule-1, and liver-type fatty acid binding protein—for the early detection of kidney damage preceding serum creatinine elevation (4), the diagnostic accuracy of these biomarkers remains limited (4, 10, 11). Consequently, further research is imperative to develop tools capable of predicting AKI at an early stage.

Machine learning, a subset of artificial intelligence, has demonstrated efficacy in predicting AKI through the development of predictive models that analyze extensive datasets pertaining to medical treatments and outcomes. The nomogram, a widely utilized visualization technique in machine learning, serves as a dependable instrument for predicting and quantifying the risk of clinical events (12, 13). While risk prediction models for AKI in cirrhotic patients with gastric variceal bleeding are relatively well-established (14, 15), there remains a notable deficiency in risk prediction models for AKI in patients experiencing upper gastrointestinal bleeding due to acute non-variceal causes. Our study identified a combination of routinely available clinical variables that could be used for the highly precise prediction of acute NVUGIB with AKI in critically ill patients.

## Materials and methods

The methodologies described in this article are consistent with the guidelines established in the Transparent Reporting of a Multivariable Prediction Model for Individual Prognosis or Diagnosis (TRIPOD) statement (16).

### Ethics approval and consent to participate

The establishment of MIMIC-IV (version 2.2) was approved by the institutional review boards of the Beth Israel Deaconess Medical Center (Boston, MA) and Massachusetts Institute of Technology (Cambridge, MA), thus, this study was granted a waiver of informed consent.

### Database

The study utilized data from the publicly available MIMIC-IV database (version 2.2), a robust critical care database situated in the United States. This database encompasses clinical information from a vast cohort of over 190,000 patients and 450,000 hospitalizations spanning the years 2008 to 2019. The data captured within the database includes a comprehensive array of patient demographics, laboratory tests, medications, vital signs, disease diagnoses, drug management, and follow-up survival outcomes.

### Participants

The study’s criteria for inclusion consisted of adult patients aged 18 years and older who were admitted to the ICU with acute NVUGIB. Exclusion criteria encompassed individuals with a baseline creatinine level suggestive of stage 5 chronic kidney disease (CKD) or those undergoing frequent renal replacement therapy.

Patients were assigned to groups utilizing a pre-seeded random number generator (123) in R software version 4.3.3, and subsequently divided into training and validation sets at a ratio of 7:3.

### Data extraction

Data was extracted from the MIMIC-IV database using PostgreSQL tools (V.1.13.1). Variables relevant to the risk of AKI were evaluated a priori, taking into consideration scientific literature, clinical significance, and predictors identified in prior studies (5, 17, 18).

For included patients, we collated data relating to clinical features as follows:

Demographic characteristics: sex, age, race.

Treatment modalities: use of diuretic use, use of aminoglycoside, use of human albumin therapy.

Comorbidities: chronic obstructive pulmonary disease (COPD), hypertension, hypotension, diabetes, heart failure, chronic liver disease, CKD, coronary and acute pancreatitis.

Laboratory test: hemoglobin, blood urea nitrogen (BUN), albumin (Alb), Serum creatinine (Scr), and glucose.

Outcome: AKI occurred during hospitalization.

For all laboratory test result parameters, we use the values at the time AKI occurred.

### Missing data handling

In the MIMIC-IV database, a noteworthy prevalence of missing data is observed. However, the exclusion of patients with incomplete data may introduce significant bias into the study. To mitigate the impact of missing data, all variables used in the analyses were thoroughly evaluated. Less than 10% of missing values were identified across all variables. Consequently, imputation was conducted by replacing missing values with means for continuous variables with normal distributions and with medians for continuous variables with skewed distributions (19). Additionally, no dichotomous variables were missing from our study.

### Definitions and outcomes

The primary outcome of interest during the ICU stay was AKI, which was defined according to the Kidney Disease Improving Global Outcomes (KDIGO) criteria (7). The use of diuretics, human albumin and aminoglycosides was categorized as any administration of these medications prior to the occurrence of AKI during the ICU stay for any indication. Hypotension was defined as any occurrence of systolic blood pressure less than 90 mmHg or diastolic blood pressure less than 60 mmHg before the onset of AKI.

### Statistical analysis

Statistical analyses were conducted using SPSS version 26.0 (IBM, Armonk, NY, USA) and R version 4.2.1. Two-sided *P*-values were employed, with statistical significance defined as *P* < 0.05. Categorical variables were expressed as percentages, while continuous variables were reported as means ± SD, medians, or ranges, depending on their normality of distribution. The chi-square test was utilized for categorical variables, while *t*-tests or Wilcoxon rank sum tests were employed for continuous variables, depending on their distributions.

To enhance the accuracy of forecasts and the comprehensibility of findings, the research employed least absolute shrinkage and selection operator (LASSO) regression analysis for variable selection and regularization (20). The variables identified in the LASSO regression model during the training phase were further examined using univariate logistic regression to determine their predictive significance for AKI (21). Variables demonstrating a *p*-value of less than 0.05 in the initial univariate logistic analyses were subsequently subjected to multivariable logistic regression analysis using a backward stepwise selection method. Additionally, the variance inflation factor (VIF) was calculated among the covariate variables in the multivariable logistic regression analysis, and VIF > 4.0 was interpreted as indicating multicollinearity. Variables with VIF > 4.0 weren’t included in the final analysis. After constructing a predictive model through multivariable logistic regression analysis, a clinical prediction nomogram and an interactive web-based application were developed utilizing Shiny apps to estimate the likelihood of AKI.

The performance of the nomogram was evaluated in both the training and validation cohorts through assessments of discrimination and calibration (22). Discrimination was measured using the C-index, which ranges from 0.5 (indicating no discrimination) to 1.0 (indicating perfect prediction), indicating the extent of predictive accuracy. Calibration was assessed by comparing predicted and actual probabilities of AKI occurrence through a visual calibration plot. Internal validation was conducted using 1000 bootstrap resamples to further evaluate the nomogram’s predictive accuracy. Additionally, a decision curve analysis (DCA), which determines the net benefit of models and predictors, was performed to assess the clinical value of the nomogram (23).

## Results

### Characteristics of patients

A total of 1,082 patients presenting with acute NVUGIB were included in the study, with 406 patients (37.5%) testing positive for AKI. The average age of the patients was 62 years, with a majority (63.1%) being male. Patients were randomly divided into training (766 patients) and validation (316 patients) cohorts. Table 1 displays the demographic and clinical characteristics of patients in each cohort. Baseline clinical features were found to be comparable between the two cohorts, with AKI rates of 38.5% and 35.1% in the training and validation cohorts, respectively.

**TABLE 1:** Characteristics of patients in the training and validation cohorts.

Characteristic	Training cohort (*n* = 766)	Validation cohort (*n* = 316)	*p-*value
**AKI, *n* (%)**			
No	471 (61.5)	205 (64.9)	0.329
Yes	295 (38.5)	111 (35.1)	
**Race, *n* (%)**			
White	500 (65.3)	208 (65.8)	0.829
Black	77 (10.1)	28 (8.9)	
Other	189 (24.7)	80 (25.3)	
**Sex, *n* (%)**			
Female	291 (38.0)	108 (34.2)	0.266
Male	475 (62.0)	208 (65.8)	
Age, years	61 (52, 73)	61 (52, 72)	0.864
**CKD, *n* (%)**			
No	716 (93.5)	286 (90.5)	0.117
Yes	50 (6.5)	30 (9.5)	
**COPD, *n* (%)**			
No	744 (97.1)	309 (97.8)	0.688
Yes	22 (2.9)	7 (2.2)	
**Coronary, *n* (%)**			
No	709 (92.6)	298 (94.3)	0.370
Yes	57 (7.4)	18 (5.7)	
**Diabetes, *n* (%)**			
No	681 (88.9)	277 (87.7)	0.631
Yes	85 (11.1)	39 (12.3)	
**Hypotension, *n* (%)**			
No	734 (95.8)	304 (96.2)	0.906
Yes	32 (4.2)	12 (3.8)	
**Hypertension, *n* (%)**			
No	586 (76.5)	247 (78.2)	0.609
Yes	180 (23.5)	69 (21.8)	
**Heart faire, *n* (%)**			
No	698 (91.1)	289 (91.5)	0.954
Yes	68 (8.9)	27 (8.5)	
**Chronic liver disease, *n* (%)**			
No	692 (90.3)	279 (88.3)	0.368
Yes	74 (9.7)	37 (11.7)	
**Use of human albumin, *n* (%)**			
No	507 (66.2)	206 (65.2)	0.807
Yes	259 (33.8)	110 (34.8)	
**Acute pancreatitis, *n* (%)**			
No	755 (98.6)	305 (96.5)	0.054
Yes	11 (1.4)	11 (3.5)	
**Use of diuretic, *n* (%)**			
No	612 (79.9)	242 (76.6)	0.257
Yes	154 (20.1)	74 (23.4)	
**Use of aminoglycosides, *n* (%)**			
No	719 (93.9)	298 (94.3)	0.892
Yes	47 (6.1)	18 (5.7)	
Hemoglobin, g/L	94 (84, 107)	94 (82, 105)	0.207
Scr (umol/L)	80 (62, 133)	80 (62, 139)	0.814
Blood urea nitrogen (mmol/L)	3.4 (2.2, 6.0)	3.7 (2.2, 6.0)	0.441
Albumin (g/L)	30 (26, 35)	30 (26, 35)	0.979
Glucose (mmol/L)	6.4 (5.3, 8.1)	6.2 (5.3, 7.9)	0.447

Scr, Serum creatinine; COPD, chronic obstructive pulmonary disease; CKD, chronic kidney disease; AKI, actue kindey injure.

### Nomogram variable screening

**FIGURE 1:**
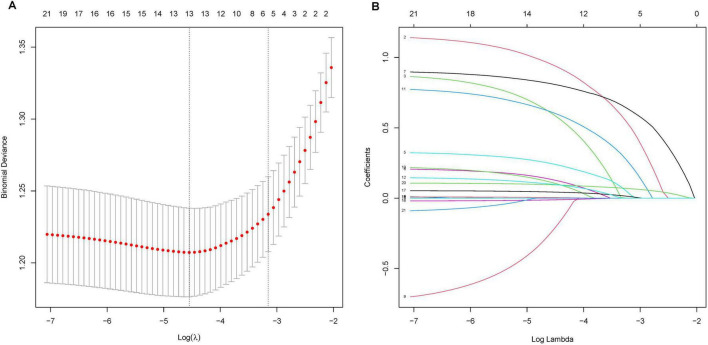
LASSO Regression Analysis for Predictor Selection. **(A)** Tuning parameter (lambda) selection in the LASSO model using 10-fold cross-validation. The x-axis represents the log(lambda), and the y-axis represents the binomial deviance. The red dots indicate the average deviance values, and the vertical bars represent the standard errors. The vertical dashed lines represent the optimal values chosen by the minimum criteria and 1 standard error of the minimum criteria (the 1-SE rule). **(B)** LASSO coefficient profiles of the 21 potential predictors. Each curve represents a predictor, with the y-axis displaying the coefficient values and the x-axis representing the log(lambda). The numbers at the top of the plot indicate the number of predictors included in the model at each log(lambda) value.

In this investigation, LASSO regression analysis was employed to identify 13 predictors with significant correlation to AKI from a pool of 21 potential predictors in the training cohort, as depicted in Figures 1A, B. The predictors associated with AKI as identified by the LASSO regression technique are detailed in Table 2 (lambda = 0.01038261). Following this, a multivariable logistic regression analysis was carried out to delve deeper into the variables that successfully passed through both univariate logistic regression and LASSO analyses.

**TABLE 2:** Univariate and multivariate logistic regression analyses of variables relating to AKI in the training cohort.

Variable	Univariate analysis		Multivariate analysis	
	OR (95% CI)	*p*-value	OR (95% CI)	*p*-value
**Race**				
White	Reference		Reference	
Black	1.03 (0.63, 1.70)	0.896	0.89 (0.51, 1.55)	0.680
Other	1.61 (1.15, 2.26)	0.006	1.40 (0.96, 2.03)	0.080
**CKD**				
No	Reference	<0.001	Reference	<0.001
Yes	4.54 (2.40, 8.57)		3.62 (1.83, 7.15)	
**Chronic liver disease**				
No	Reference	<0.001	Reference	0.003
Yes	2.92 (1.78, 4.79)		2.27 (1.33, 3.88)	
**Use of human albumin**				
No	Reference	<0.001	Reference	<0.001
Yes	3.18 (2.33, 4.35)		2.45 (1.76, 3.42)	
**Acute pancreatitis**				
No	Reference	0.445	NA	
Yes	0.59 (0.16, 2.26)			
**COPD**				
No	Reference	0.019	Reference	0.080
Yes	2.88 (1.19, 6.96)		2.39 (0.90, 6.31)	
**Hypertension**				
No	Reference	0.017	Reference	0.315
Yes	1.51 (1.08, 2.12)		1.22 (0.83, 1.81)	
**Diabetes**				
No	Reference	0.002	Reference	0.221
Yes	2.05 (1.30, 3.22)		1.40 (0.82, 2.41)	
**Use of diuretic**				
No	Reference	0.007	Reference	0.297
Yes	1.64 (1.15, 2.34)		1.24 (0.83, 1.86)	
Hemoglobin	0.91 (0.76, 1.10)	0.348	NA	
Blood urea nitrogen	1.81 (1.51, 2.15)	<0.001	1.48 (1.23, 1.78)	<0.001
Albumin	0.76 (0.63, 0.93)	0.006	0.86 (0.70, 1.07)	0.177
Glucose	1.23 (1.08, 1.40)	0.002	1.21 (1.06, 1.39)	0.005

Based on the findings of the stepwise logistic regression analysis, the model incorporating five independent predictors of AKI, including CKD, use of human albumin, chronic liver disease, glucose, and blood urea nitrogen, demonstrated the lowest AIC value within the training cohort. Additionally, the VIF values for all variables were less than 4, suggesting the absence of collinearity among the screened predictors (Table 2).

### Nomogram construction and performance in the training cohort

**FIGURE 2:**
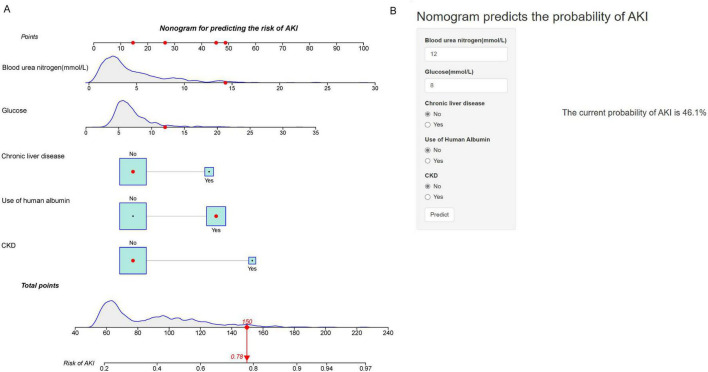
Nomogram for Predicting the Risk of AKI. **(A)** Nomogram to predict the risk of AKI in patients with acute NVUGIB. The nomogram integrates multiple predictors, including blood urea nitrogen (mmol/L), glucose (mmol/L), chronic liver disease, use of human albumin, and CKD. Each predictor has a corresponding point scale, which is used to calculate the total points. The total points are then used to determine the risk of AKI, displayed on the bottom scale. **(B)** Web-based calculator interface for predicting AKI risk. Users input values for blood urea nitrogen, glucose, chronic liver disease, use of human albumin, and CKD status to obtain the predicted probability of AKI. The example provided shows a current probability of AKI at 46.1%.

Utilizing the outcomes of multivariate Logistic regression analysis, a nomogram (Figure 2A) was developed to visually represent a model incorporating independent predictors. For instance, a patient presenting with a blood urea nitrogen level of 14.3 mmol/L, glucose level of 12 mmol/L, absence of chronic liver disease and AKD, and using human albumin, possesses a current AKI risk score of 150, which equates to a 78% probability of developing AKI. This nomogram can be accessed online at https://risk-prediction-model-web-calculator20240327.shinyapps.io/AKI_probability_of_UBG/, as depicted in Figure 2B. Users are required to interact with the in-line graph by selecting either “Yes” or “No” from the provided options, inputting pertinent laboratory test results, and subsequently choosing “Predict” to ascertain the probability of AKI occurrence during the patient’s ICU admission.

**FIGURE 3:**
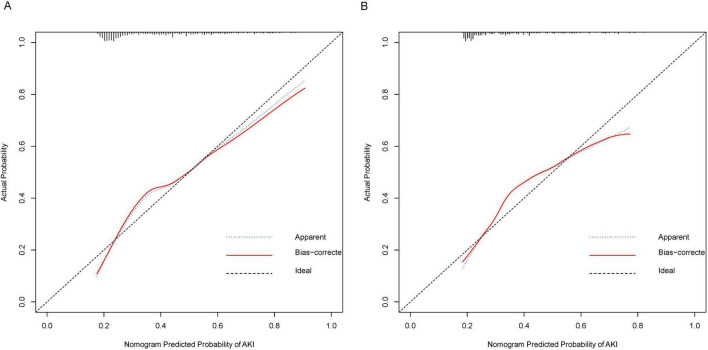
Calibration Curves for Nomogram Predicted Probability of AKI. These calibration plots compare the predicted probabilities of AKI against the actual observed probabilities, demonstrating the accuracy of the nomogram model. **(A)** Calibration curve for the training cohort. **(B)** Calibration curve for the validation cohort. The x-axis represents the nomogram predicted probability of AKI, while the y-axis shows the actual probability. The plot includes the apparent calibration (blue dotted line), the bias-corrected calibration (red solid line), and the ideal calibration (black dashed line). The closer the red line is to the black line, the more accurate the model.

The C-index of the nomogram was calculated to be 0.73 (95% CI: 0.70–0.77) for the training cohort. The calibration curve illustrated in Figure 3A exhibits a satisfactory concordance between the anticipated and actual occurrences for the likelihood of AKI in the training cohort. The lack of statistical significance (*P* = 0.580) in the Hosmer–Lemeshow test implies that the model did not demonstrate overfitting.

### External validation of the nomogram 2 in the validation cohort

In the validation cohort, the nomogram exhibited a C-index of 0.72 (95% CI 0.66–0.79) for the assessment of AKI risk. Additionally, a well-calibrated risk estimation was demonstrated through the calibration curve (Figure 3B).

### Clinical value of the nomogram

**FIGURE 4:**
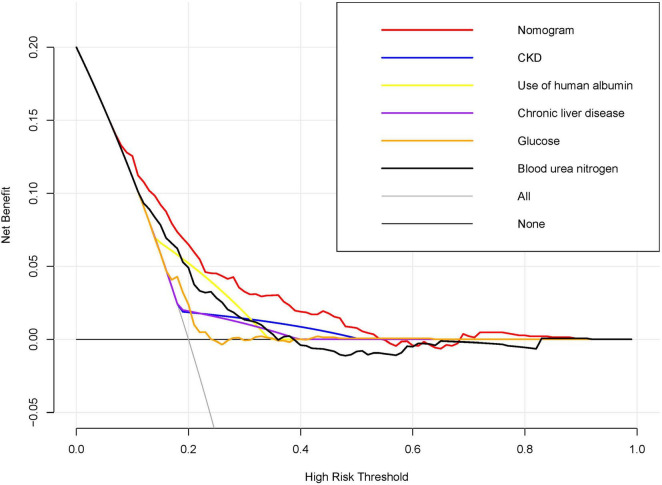
Decision Curve Analysis for the Nomogram Predicting AKI in acute NVUGIB Patients. Decision curve analysis was conducted to compare the net benefits of the nomogram against individual predictors across a spectrum of high-risk thresholds. The x-axis denotes the high-risk threshold for predicting acute kidney injury (AKI), while the y-axis indicates the net benefit. The red line, representing the nomogram, demonstrates superior net benefit across most thresholds when compared to individual predictors, including chronic kidney disease (CKD), use of human albumin, chronic liver disease, glucose levels, and blood urea nitrogen.

Figure 4 illustrates the outcomes of decision curve analysis for the nomogram, highlighting the high-risk threshold probability at which a clinician can assess a patient’s risk of AKI and the potential advantages of intervention. The decision curve indicates that employing the nomogram for AKI prediction can yield substantial benefits when a clinician’s threshold probability falls between 7% and 54%, with the nomogram exhibiting greater predictive accuracy compared to a single predictor within this specified range.

## Discussion

AKI is a serious complication in patients with acute NVUGIB significantly impacting morbidity and mortality. In this study, we developed a nomogram to quantitatively predict the risk of AKI in patients with acute non-variceal UGIB based on patient-specific factors. This predictive tool can be utilized to assess individual AKI risk, thereby facilitating personalized treatment and surveillance strategies. The significance of the present study lies in the development of the nomogram utilizing a substantial cohort of ICU patients diagnosed with acute non-variceal UGIB. Furthermore, the nomogram’s performance underwent rigorous assessment and internal validation.

The current AKI diagnostic criteria, established by KDIGO in 2012, rely on changes in serum creatinine and urine output, which can miss early or subclinical AKI, depend on baseline creatinine levels that may not always be known, and are influenced by non-renal factors like hydration and muscle mass. Consequently, research efforts have been directed towards identifying susceptibility and exposure factors associated with AKI to facilitate preemptive preventive measures. These measures include optimizing fluid management, avoiding nephrotoxic medications, closely monitoring renal function in high-risk patients, and using alternative imaging methods to minimize contrast exposure. Nonetheless, the onset and progression of AKI involve intricate pathophysiological mechanisms, rendering the accurate assessment of AKI risk based on individual susceptibility and exposure challenging. This complexity often leads to the potential for over-treatment in preventive strategies. Another particular strength of this study is the consideration of a range of previously reported clinical features and laboratory findings related to AKI (5, 7, 17, 18, 24). In the present study, we similarly noted that CKD, glucose, chronic liver disease were closely related to AKI in patients with acute NVUGIB, which is consistent with most studies on variceal upper gastrointestinal bleeding (20–23). In contrast to variceal upper gastrointestinal bleeding, where albumin administration is generally advantageous for the prevention of AKI, our study identified a positive correlation between albumin use and the risk of AKI in patients with acute NVUGIB. This association may be attributable to enhanced albumin filtration and modified tubular albumin uptake in these individuals. Nonetheless, the relationship between human blood albumin administration and the incidence of AKI remains a subject of ongoing debate, (25–27), and the precise underlying mechanism remains uncertain. It is worth mentioning that we found that blood urea nitrogen was more predictive of AKI than Scr in patients with acute UGIB. This disparity may be attributable to fasting-induced alterations in muscle metabolism and renal blood flow, which influence Scr production and excretion. Despite these physiological changes, Scr levels tend to remain relatively stable and are less affected by short-term dietary modifications. Conversely, blood urea nitrogen levels are more susceptible to variations in protein catabolism and renal perfusion, rendering blood urea nitrogen a more sensitive marker for detecting changes in renal function and assessing the risk of AKI in this patient population.

The capacity to precisely forecast the incidence of AKI in patients experiencing acute NVUGIB holds substantial importance, given that AKI represents a heterogeneous syndrome necessitating individualized care and management approaches (28). In contrast to the population-based or large cohort data utilized by KDIGO clinical practice guidelines, nomograms offer a more individualized approach to delivering prognostic information to patients. To the best of our knowledge, this study represents the first attempt to develop an AKI risk prediction model that independently evaluates previously proposed risk variables for their inclusion in a formal nomogram specifically for patients with acute NVUGIB. Most studies on acute NVUGIB primarily concentrate on assessing the severity of bleeding, patient prognosis, and the risk of rebleeding. For instance, the Rockall score is employed to determine the necessity for further endoscopic intervention, while the Forrest score is utilized to evaluate the risk of endoscopic rebleeding (29). However, AKI, a complication associated with more severe short-term and long-term prognoses, is frequently overlooked. The lack of timely and effective interventions for AKI significantly exacerbates the complexity and cost of treatment. Therefore, we utilized clinically accessible laboratory results and assessed patients’ susceptibility to AKI to develop a nomogram aimed at providing individualized AKI risk predictions for patients with acute NVUGIB in the intensive care unit. This approach aligns with the contemporary emphasis on personalized medicine.

The primary and ultimate justification for employing the nomogram lies in its capacity to assess the necessity for individualized supplementary treatment or care. Nevertheless, the metrics of risk-prediction performance, including discrimination and calibration, fail to encapsulate the clinical implications associated with specific levels of discrimination or degrees of miscalibration (30–32). Therefore, to substantiate the clinical utility of our nomogram, we undertook an evaluation to ascertain whether decisions informed by the nomogram would result in improved patient outcomes. Considering the inherent difficulties of executing a multi-institutional prospective validation, particularly due to the complexities involved in aggregating clinical data from multiple institutions, we opted to utilize decision curve analysis as an alternative methodological approach in this study. This study introduces an innovative methodology for evaluating the clinical implications of decisions grounded in threshold probability, thereby facilitating the calculation of net benefit (16, 33). The decision curve analysis conducted herein reveals that employing a nomogram for the prediction of AKI yields greater benefits when the threshold probability for physicians between 7% and 54%, as opposed to the strategies of treating all patients or treating none.

This study has limitations. First, its monocentric design within a single ICU, which restricts the generalizability of the findings and the dynamic online nomogram to other centers or countries. Further research is needed to validate the model in varied settings. Second, the MIMIC database’s lack of novel biomarkers, such as cystatin C, neutral gelatin-associated lipocalcin, NT-proBNP, uNGAL, and uAGT, precluded us from enhancing the model’s predictive capacity. Third, the retrospective design of the study inherently limited our ability to eliminate bias. However, rigorous inclusion criteria were applied to ensure that both the control and case groups accurately reflected real-world conditions.

## Conclusion

Our study presents an innovative online nomogram that incorporates clinical risk factors to facilitate personalized prediction of AKI in patients with acute NVUGIB upon ICU admission. This predictive tool holds significant potential in identifying acute NVUGIB patients who are most likely to benefit from targeted interventions for the prevention and management of AKI.

## Data Availability

The raw data supporting the conclusions of this article will be made available by the authors, without undue reservation.
