# Corrigendum: Development and validation of a web-based nomogram for acute kidney injury in acute non-variceal upper gastrointestinal bleeding patients

**DOI:** 10.3389/fmed.2025.1593738

**Published:** 2025-03-26

**Authors:** Chaolian Wei, Honghua Cao, Lina Huang, Lu-Huai Feng

**Affiliations:** ^1^Department of Gastric and Abdominal Tumor Surgery, Guangxi Medical University Cancer Hospital, Nanning, China; ^2^Department of Gastrointestinal Surgery, Guigang People's Hospital, Guigang, China; ^3^Department of Endocrinology and Metabolism Nephrology, Guangxi Medical University Cancer Hospital, Nanning, China

**Keywords:** acute kidney injury, prediction, nomogram, intensive care unit, upper gastrointestinal bleeding

In the published article, there was an error in affiliation(s) [1 and 3]. Instead of “[The Affiliated Tumor Hospital of Guangxi Medical University]”, it should be “[Guangxi Medical University Cancer Hospital]”.

The authors apologize for this error and state that this does not change the scientific conclusions of the article in any way. The original article has been updated.

